# Genetic relationship and diversity in a sesame (*Sesamum indicum L*.) germplasm collection using amplified fragment length polymorphism (AFLP)

**DOI:** 10.1186/1471-2156-7-10

**Published:** 2006-02-16

**Authors:** Hernán E Laurentin, Petr Karlovsky

**Affiliations:** 1 Biologic Sciences Department, Agronomy Faculty, Universidad Centroccidental Lisandro Alvarado, Barquisimeto, Venezuela; 2 Institute of Phytopathology and Plant Protection, Georg-August-University, 37077 Goettingen, Germany

## Abstract

**Background:**

Sesame is an important oil crop in tropical and subtropical areas. Despite its nutritional value and historic and cultural importance, the research on sesame has been scarce, particularly as far as its genetic diversity is concerned. The aims of the present study were to clarify genetic relationships among 32 sesame accessions from the Venezuelan Germplasm Collection, which represents genotypes from five diversity centres (India, Africa, China-Korea-Japan, Central Asia and Western Asia), and to determine the association between geographical origin and genetic diversity using amplified fragment length polymorphism (AFLP).

**Results:**

Large genetic variability was found within the germplasm collection. A total of 457 AFLP markers were recorded, 93 % of them being polymorphic. The Jaccard similarity coefficient ranged from 0.38 to 0.85 between pairs of accessions. The UPGMA dendrogram grouped 25 of 32 accessions in two robust clusters, but it has not revealed any association between genotype and geographical origin. Indian, African and Chinese-Korean-Japanese accessions were distributed throughout the dendrogram. A similar pattern was obtained using principal coordinates analysis. Genetic diversity studies considering five groups of accessions according to the geographic origin detected that only 20 % of the total diversity was due to diversity among groups using Nei's coefficient of population differentiation. Similarly, only 5% of the total diversity was attributed to differences among groups by the analysis of molecular variance (AMOVA). This small but significant difference was explained by the fact that the Central Asia group had a lower genetic variation than the other diversity centres studied.

**Conclusion:**

We found that our sesame collection was genetically very variable and did not show an association between geographical origin and AFLP patterns. This result suggests that there was considerable gene flow among diversity centres. Future germplasm collection strategies should focus on sampling a large number of plants. Covering many diversity centres is less important because each centre represents a major part of the total diversity in sesame, Central Asia centre being the only exception. The same recommendation holds for the choice of parents for segregant populations used in breeding projects. The traditional assumption that selecting genotypes of different geographical origin will maximize the diversity available to a breeding project does not hold in sesame.

## Background

Sesame (*Sesamum indicum *L.) is one of the most ancient crops [1]. It is grown in tropical and subtropical areas [2] on 6.5 million hectares worldwide, producing more than three million tons of seed [3]. India, Sudan, Myanmar and China are the most important sesame producers with 68 % of the world production. Sesame seed, which is highly nutritive (50% oil and 25% protein), is traditionally used for direct consumption and as a source of oil of excellent quality due to the presence of natural antioxidants such as sesamin and sesamol [4]. Potentially beneficial effects of sesame on human health have recently renewed the interest in this ancient crop.

Despite the nutritional value and historic and cultural importance of sesame, the research on sesame has been scarce. For example, no international CGIAR (Consultative Group on International Agricultural Research) agency is mandated to study sesame [5]. Information on the genetic diversity in sesame is limited as well. Sesame diversity centres have been identified as India, China, Central Asia, Near East and Abysinia in classical studies [6,7]. More recently, a high level of variability of morphological characters within different sesame collections was reported [8,9]. Genetic variability in sesame has also been studied by molecular techniques, including isozymes [10,11], RAPD [12,13] and ISSR [14]. Amplified fragment length polymorphism (AFLP) has only been used in linkage analysis [15]. AFLP is a promising technique for the characterization of genetic diversity in sesame because it possesses a high degree of reproducibility and discriminatory power [16]. It has been successfully applied to many cultivated and wild plants, including faba bean (*Vicia faba *L.) [17], grapevine (*Vitis vinifera *L.) [18], adzukibean (*Vigna angularis *Willd.) [19], squash (*Cucurbita pepo *L.) [20], *Nicotiana attenuata *[21], plantain (*Musa *spp.) [22], sorghum (*Sorghum bicolor *L.)[23], alfalfa (*Medicago sativa *L.) [24] wheat (*Triticum turgidum *L. subsp. Durum (Desf.) Husn) [25], and coffee (*Coffea arabica *L.) [26].

The aim of the present study was to clarify genetic relationships among 32 sesame accessions from the Venezuelan Germplasm Collection, which represents genotypes from 5 geographical regions, and to determine the relationship between geographical distribution and genetic diversity.

## Results

### AFLP results

A total of 457 AFLP markers were recorded using 8 primer combinations on 32 sesame accessions. Ninety-three percent of markers were polymorphic (Table 1). Fifty-nine percent of the markers ranged from 100 to 300 nucleotides in size. Forty-seven bands (10.3 %) were unique, 25 belonging to African accessions, 10 to Indian accessions, 8 to China-Korean-Japan accessions, 3 to Central-Asian accessions and 1 to Western-Asian accession.

**Table 1:** List of primer combinations used in the present study and some characteristics of the amplification products.  Bands were considered polymorphic if the frequency of one of its states (present or absent) is less or equal to 0.97 (present or absent in at least 31 from 32 accessions)

Primer combination	Total number of bands	Polymorphic bands	% of polymorphic bands
(Cy5)E_ACA+M_CTCA	48	43	90
(Cy5)E_ACA+M_CAA	68	66	97
(Cy5)E_ACA+M_CCA	52	52	100
(Cy5)E_ACA+M_CGAA	45	38	84
(Cy5)E_ACA+M_CAT	93	86	92
(Cy5)E_ACA+M_CAG	57	56	98
(Cy5)E_ACA+M_CCC	56	52	93
(Cy5)E_ACA+M_CAC	40	32	83

### Phenetic analysis

Jaccard's similarity coefficients ranged from 0.38 (between one accession from India and one from Korea) to 0.85 (between one accession from Turkey and one from Syria), with an average of 0.65. Within each geographical region sampled similarity coefficients were 0.59 for Africa, 0.61 for China-Korea-Japan, 0.63 for India, 0.68 for Western Asia and 0.80 for Central Asia.

**Figure 1:**
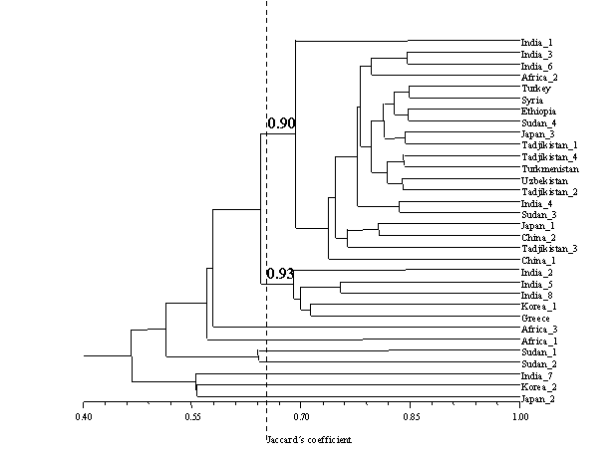
**Dendrogram for 32 sesame accessions (cophenetic correlation 0.95). **Values from bootstraping analysis are indicated. Two groups are clearly identified, and these nodes have a similarity average and standard deviation of 69.2 ± 2.6 (the upper) and 69.0 ± 1.2. (the lower)

**Figure 2:**
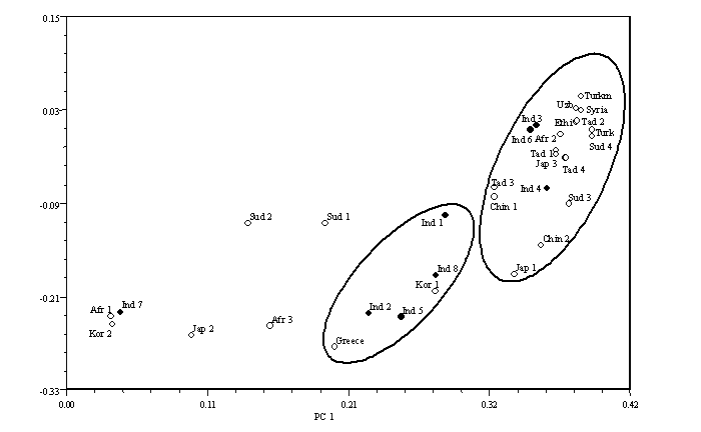
Biplot of principal coordinates analysis for 32 sesame accessions.

Figure 1 displays a UPGMA dendrogram obtained using similarity coefficients. Two robust groups were identified at a similarity value of 0.65 by bootstraping (bootstrap values 90% and 93%). These clusters included 25 of 32 accessions used in the analysis. The cophenetic correlation coefficient (0.95) indicated little distortion between the original similarity values from the similarity matrix and the values used to construct the dendrogram. Furthermore, the standard deviation for the two main clusters was less than 4% (see legend to Figure 1). Figure 2 displays the location of the same 32 accessions on a bidimensional space of principal coordinates analysis, which represented 74 % of total variation among accessions.

### Genetic diversity

Table 2 summarises estimated Nei's parameters related to genetic diversity, showing that only 20% of the total variation in allele frequencies corresponds to differences among groups. Average diversity within groups (H_ST_) ranged between 0.14 for Central Asia and 0.21 for Africa. Genetic distances among groups are very low (Table 3). Central Asia accessions had the lowest probability of sharing the same allele frequencies in all sampled loci with the other geographical regions.

**Table 2:** Polymorphic loci and genetic diversity of five groups of sesame accessions, according their geographical distribution

Population	Polymorphic loci	%	Hs
India	313	63	0.1957
Africa	352	71	0.2129
Western Asia	187	38	0.1516
Central Asia	199	40	0.1445
China-Korea-Japan	292	59	0.1916
		Average Hs = 0.1793± 0.0266	
		Ht = 0.2244 ± 0.0325	
		Gst = 0.2013	

**Table 3:** Unbiased measures of identity and genetic distance (Nei, 1978) among groups of sesame accessions. Nei's genetic identity is shown above diagonal, genetic distance below diagonal.

	India	Africa	Western Asia	Central Asia	China-Korea-Japan
India		0.978	0.956	0.925	0.979
Africa	0.023		0.950	0.933	0.989
Western Asia	0.045	0.051		0.928	0.951
Central Asia	0.078	0.069	0.075		0.926
China-Korea-Japan	0.021	0.011	0.051	0.077	

### Analysis of molecular variance

Table 4 shows the analysis of molecular variance. It indicates that 5% of the variance among the 32 AFLP patterns was due to differences among groups and 95% was due to differences within groups. However, despite the small value for variation among groups, it was statistically significant (P < 0.05). Table 5 displays that this finding can be explained by a large difference between the variation of Central Asia accessions with respect to the other diversity centres, since all of them, and only they, were significant (P < 0.05).

**Table 4:** AMOVA for the partitioning AFLP variation in sesame

Source of variation	Degree of freedom	SS	CV	% total	p
Among geographical areas	4	328.37	3.32	5.14	0.02
Within geographical areas	27	1654.47	61.28	94.86	
Total	31	1982.84			

Fixation index (Fst) = 0.0514					

**Table 5:** Pairwise comparison of groups of sesame accessions by AMOVA. Genetic distance (F_ST_) between groups of sesame accessions is shown below diagonal. Probability of random distance (F_ST_) larger than the observed distance after 1000 permutations is shown above diagonal.

	India	Africa	China-Korea-Japan	Western Asia	Central Asia
India		0.161	0.233	0.367	0.005
Africa	0.027		0.455	0.540	0.008
China-Korea-Japan	0.014	-0.003		0.301	0.005
Western Asia	0.000	-0.022	0.014		0.012
Central Asica	0.141	0.098	0.132	0.103	

## Discussion

*Sesamum indicum *L. has a large genetic variability, which should be taken into account when planning conservation strategies or when sesame variability is used in breeding programs. This high level of polymorphism in sesame has been reported before for its morphology [8,9]. Early molecular studies have not confirmed this. Isozyme studies concluded that cultivated sesame has a narrow genetic base [10,11], However, the number of loci sampled in these studies were limited and enzymes represent merely coding sequences. Furthermore, all synonymous and many non-synonymous mutations are not detected with isozyme analysis [27].

A RAPD-based study on sesame carried out on 36 Indian accessions and 22 accessions from other countries [12] and a study on a Turkish sesame collection [13] concluded that sesame has a high level of genetic variability. An ISSR-based study on Korean accessions and cultivars from 12 countries found a low level of polymorphism in this particular marker, but the authors cautioned that their method had low resolution and problems with visualization [14]. Generally, methods based on arbitrarily primed PCR tend to reveal a higher degree of genetic variability as compared to other methods [28].

Figures 1 and 2 allow assessment of the genetic relationship among accessions. There is no obvious relationship between geographical origin and grouping based on genetic similarities. Particularly Indian, African and Chinene-Japanese-Korean accessions are distributed throughout clusters in UPGMA analysis and the whole two-dimensional space in PCA. Nei's parameters displayed in Table 2 and 3, especially the G_ST _value of 0.20, support the lack of association between geographical origin and population differentiation. Due to AFLP are dominant markers, heterozygocity cannot be directly observed. Therefore three methods are available for the calculation of allele frequencies from dominant marker data: a classical approach based on Hardy-Weinberg assumption, which we used in this work (see Material and Methods for details), a modification of the classical approach by the exclusion of loci with three or less recessive homozygotes [29], and a Bayesian approach [30]. Comparison of allele frequencies calculations from these three methods on AFLP data resulted essentially identical; furthermore potential biases in the estimation of null allele frequency are largely eliminated in highly polymorphic dominant marker data [31]. Confidence in our calculations of Nei's parameters are supported by this finding.

However, the interpretation of results obtained using Nei's parameters should be used cautiously. H_T _is called "average heterozygosis" when it is calculated from data on co-dominant markers and "average genetic diversity" or "heterogeneity" when it is calculated from data on dominant markers. Heterogeneity values might overestimate the number of real loci, whereas the number of alleles per locus is underestimated. Thus, heterogeneity measures have only relative value and cannot be compared with values obtained from other molecular markers [32]. Additionally, reliable estimation of average heterozygosis with small number of individual are based on large number of loci (>50) and low average heterozygosis (<0.1) [33]. Our results indicate a heterogeneity higher than 0.1, however, we are more interested in the partition of variation (within and between groups of accessions) than in the value by itself.

AMOVA results shown in Table 4, support the lack of association between geographical origin and population differentiation as well. AMOVA reported differences among geographical regions, which were significant but represented only 5% of the total variation. Table 5 shows that the differences between Central Asian accessions and the other centres were responsible for this part of the variation. These results, together with the fact that all Central Asian accessions were grouped in one cluster, indicate a narrow variation within this centre as compared to the variance in AFLP patterns of the whole germplasm collection. Furthermore, Central Asian accessions had the lowest H_ST _value (0.14) though it had more polymorphic loci than Western Asia accessions. A possible explanation of this result is that allele frequencies in Central Asia were close to 0 or 1, which could be a consequence of genetic drift. If a strong genetic flow was the cause of the lack of association between geographical origin and genetic differences in sesame, the genetic flow to Central Asia in recent time was limited.

This lack of association between geographical distribution and classification based on molecular markers in sesame was explained by the exchange of sesame among widely separated locations [14]. However, this study used predominantly commercial cultivars, which resulted from a systematic selection process on material of unreported origin, while the origin of material used in our study is known.

The genetic variability in Indian accessions of sesame is high [6], as shown both for molecular [12] and morphological markers [9]. Sesame seems to have been domesticated in India [5], which could explain the high genetic variability among Indian accessions. In our study, African and Chinese-Korean-Japanese accessions showed as high a genetic variability as Indian accession.

Africa has also been considered the origin of sesame [2,34], based on the fact that most of the wild *Sesamum *species are endemic there. Reports on the variability of cultivated sesame in Africa are controversial, claiming both low [12] and high [[35] and our results] level of variability. Some authors consider Abysinia (Ethiopia) as the basic diversity centre for sesame [6,7].

Accessions from China, Korea and Japan, which we grouped into one diversity centre, have been studied separately by some authors. A unique allele was found in Chinese-Japanese accessions in a study on isozymes [11]. China is considered a secondary centre of diversity for sesame [7]. Genetic variability among Korean landraces is higher than among Korean cultivars [14].

Central Asia and Western Asia accessions had the lowest genetic variability in our study. Reports on the genetic variability of Western Asia accessions are scarce and no information has been published so far about Syrian and Greek sesame. Turkish landraces have been compared to each other with RAPD recently [13], but the relationship to accessions from other geographical region has not been investigated. We analysed only three accessions from Western Asia. Remarkably, the highest similarity found among all 32 accessions corresponded to similarity values between two of these accessions (one from Syria and one from Turkey). The third accession was not closely related to these.

Distribution of genetic diversity in a plant species depends on its evolution and breeding system, ecological and geographical factors and often on human activities [36]. Cross-pollination may play a role, because it can reach up to 60% in sesame, depending on the presence of suitable insects at flowering time [12]. Ecological and geographical factors obviously have not played an important role in the evolution of sesame since we have not found any association between genetic diversity and accession origin.

Sesame growers have been manipulating the crop due to migration and trade for centuries, causing a steady gene flow among different geographical areas. The oldest remnants of sesame, found in the Harappa valley in the Indian subcontinent [1], date the origin of these activities to at least 5500 BP. Therefore, we believe that the most important factor affecting the current genetic structure in sesame were human activities.

## Conclusion

AFLP analysis revealed a high degree of genetic polymorphism in sesame accessions within all diversity centres except Central Asia. Phenetic analysis has not shown any association between geographic origin and AFLP patterns. According to Nei's diversity indexes, 80 % of the total genetic diversity in sesame is represented within diversity centres. This result was corroborated by analysis of molecular variance (AMOVA) which indicated that 95 % of the variation among accessions were due to variation within diversity centres. These results suggest that conservation strategies do not need to cover all diversity centres as long as they sample a sufficient number of accessions. Similarly, choosing parent genotypes for breeding programs from many diversity centres as compared to sampling just one centre (except Central Asia) is not likely to increase the variability among progeny significantly. Regardless of how many diversity centres are sampled, both conservation strategies and breeding programs would benefit from using AFLP or another genome fingerprinting technique to maximise the genetic variability covered by the selected genotype set.

## Methods

### Plant material

Thirty-two accessions from Centro Nacional de Investigaciones Agropecuarias (CENIAP) Germplasm Bank (Table 6) were grown in the greenhouse. These accessions originate from five different geographical regions representing the proposed diversity centres for sesame [6,7], and the geographical areas included in the germplasm bank; they were chosen randomly within each geographical region, using more accessions for the two proposed origin centres (India and Africa). The accessions were grouped into one of the following diversity centres: India, Africa, China-Korea-Japan, Central Asia and Western Asia.

**Table 6:** Accessions from CENIAP Germplasm Bank (Venezuela) and their respective origin country and diversity centre

**Accessions**	**Country of Origin**	**Working code**	**Diversity Centre**
93-2223	India	India 1	India
95-465	India	India 2	India
95-469	India	India 3	India
95-447	India	India 4	India
89-007	India	India 5	India
93-2224	India	India 6	India
95-464	India	India 7	India
92-2918	India	India 8	India
92-3091	Korea	Korea 1	China-Japan-Korea
92-3093	Korea	Korea 2	China-Japan-Korea
92-2922	Turkey	Turkey	Western Asia
92-3125	Greece	Greece	Western Asia
93-2022	Syria	Syria	Western Asia
93-2113	Sudan	Sudan 1	Africa
92-310	Sudan	Sudan 2	Africa
93-2010	Ethiopia	Ethiopia	Africa
95-272	Unknown	Africa 1	Africa
92-2872	Sudan	Sudan 3	Africa
93-2105	Sudan	Sudan 4	Africa
95-234	Unknown	Africa 2	Africa
95-223	Unknown	Africa 3	Africa
92-2856	Japan	Japan 1	China-Japan-Korea
92-3030	Japan	Japan 2	China-Japan-Korea
92-3031	Japan	Japan 3	China-Japan-Korea
92-3108	China	China 1	China-Japan-Korea
95-383	China	China 2	China-Japan-Korea
92-2930	Tadjikistan	Tadjikistan 1	Central Asia
92-2947	Uzbekistan	Uzbekistan	Central Asia
92-2952	Turkmenistan	Turkmenistan 1	Central Asia
92-2955	Turkmenistan	Turkmenistan 2	Central Asia
92-2950	Tadjikistan	Tadjikistan 2	Central Asia
92-2917	Tadjikistan	Tadjikistan 3	Central Asia

### DNA extraction

Three grams of apical young leaves from 6 plants per accession were collected and used for DNA extraction. Leaves were ground in liquid nitrogen and tissue powder was dispersed in CTAB buffer (2.3 g sorbitol, 1 g N-laurylsarcosine, 0.8 g CTAB, 4.7 g sodium chloride, and 1 g polyvinylpolypyrolidone in total volume of 100 ml of 20 mM EDTA, 10 mM Tris, pH set to 8.0) containing 0.4 mg proteinase K and 20 μL mercaptoethanol. The homogenates were incubated for 10 min at 42°C and 10 min at 65°C, cooled to room temperature and extracted with 8 ml of chloroform-isoamylalcohol (24:1). Phases were separated by centrifugation for 10 min at 12000 RCF (relative centrifugal force or g value). Polyethyleneglycol (PEG6000, SERVA Electrophoresis, Germany) stock solution (30%) was added to the aqueous phase to a final concentration of 6 %, mixed, and after 30 min of incubation at room temperature the precipitated DNA was sedimented by centrifugation for 20 min at 12,000 RCF. Pellets were washed twice with 70% ethanol and dissolved in 200 μL TE buffer (10 mM Tris/HCl pH 8.0, 0.1 mM EDTA). 500 μL of 5 M ammonium acetate solution were added and samples were kept at 0°C for 30 min, centrifuged for 30 min at 4°C and 18000 RCF. 500 μL of isopropanol were added to the supernatant and DNA was precipitated for 10 min at room temperature. Samples were centrifuged at 18000 RCF at room temperature for 10 min; pellets were washed twice with 70% ethanol, dried and dissolved in 200 μL of TE buffer. DNA concentration was determined by electrophoresis in a 0.8 % agarose gel with lambda DNA standard.

### AFLP analysis

AFLP analysis was performed as originally proposed [37] with minor modifications [38]. 250 ng of DNA were used for each reaction. DNA was digested with 10 U EcoRI and 3 U of Tru1I (both entzymes from MBI Fermentas, Germany) in buffer recommended by the manufacturer in a total volume of 15μl at 37°C for 90 min, followed by 90 min at 65°C. 10 μl of a solution with a final concentration of 5 pmol of EcoRI adapter, 50 pmol of Tru1I adapter, 1× T4 DNA ligase buffer and 1U T4 DNA ligase (MBI Fermentas, Germany) were added to the digested DNA. The solution was incubated at 20°C for 2 h, T4 ligase was inactivated by heating to 65°C for 10 min and the mixture was diluted 10-fold with TE buffer. Following ligation, a first amplification was carried out with primers containing one selective nucleotide (cytocine and adenine for MseI and EcoRI primers, respectively) (Table 7), dNTPs (0.125 mM, Takara Bio Inc., Japan), 1× PCR buffer (MBI Fermentas, Germany), 1.5 mM MgCl_2 _and 1 U Taq polymerase (MBI Fermentas, Germany) were added in a total volume of 10 μl. PCR was performed for 20 cycles, which consisted of 30 s at 94°C, 1 min at 56°C and 1 min at 72°C in a Tpersonal thermocycler (Biometra, Göttingen, Germany). The PCR products were diluted 10-fold with TE buffer. The second amplification was carried out with eight primer combinations using labelled EcorRI-primer (Cy5)E_ACA combined with one of the eight MseI primers listed in Table 7. The PCR mixture consisted of 2 μL of diluted preamplified DNA, 4.2 ng of (Cy5)E_ACA primer, 11.4 ng of MseI primer 0.25 mM, dNTPs (Takara Bio Inc., Japan), 1× R buffer (MBI Fermentas, Germany), 1.5 mM MgCl_2 _and 1 U Taq polymerase (MBI Fermentas, Germany) in a total volume of 10 μL. The thermocycler program consisted of two segments. The first segment comprised 12 cycles with the annealing temperature decreased from 65°C by 0.7°C in each cycle: 30 s at 94°C, 30 s at 65°C to 57.3°C and 1 min at 72°C. The second segment consisted of 23 cycles of 30 s at 94°C, 1 min at 56°C and 1 min at 72 °C. The PCR products were mixed with 10 μL of loading buffer (98 % formamide, 10 mM EDTA and 0.025 % bromophenolblue), denatured for 4 minutes at 90°C and 5 μL of this mixture were loaded onto a 7 % polyacrylamide gel ReproGel™ LongRead (Amersham Pharmacia Biotech, Uppsala, Sweden) in an ALFexpress II DNA analyser (Amersham Pharmacia Biotech, Uppsala, Sweden). Three microliters of Genemark 500 Fluorescent DNA ladder labeled with Cy5 (Northernbiothech, Weston, USA) were loaded on each gel and the electrophoresis was performed for 700 min at 1500 V, 25 W, 60 mA and 55°C. The chromatogram recorded by software ALFwin™ Sequence Analyser 2.00 (AmershamPharmacia Biotech, Uppsala, Sweden) was transformed to a pseudogel image in TIFF-format, visualized in Adobe^R ^ImageReady™ version 3.0 (Adobe Systems Inc., USA) and analyzed using GelComparII (Applied Maths, Belgium).

**Table 7:** Primer sequences used in preamplification and amplification

**Primer**	**Sequence **
**Primer**	**Sequence **
(Cy5)AFLP_E_ACA	(Cy5)GACTGCGTACCAATTCACA
AFLP_M_C	GATGAGTCCTGAGTAAC
AFLP_M_CAA	GATGAGTCCTGAGTAACAA
AFLP_M_CAT	GATGAGTCCTGAGTAACAT
AFLP_M_CAG	GATGAGTCCTGAGTAACAG
AFLP_M_CAC	GATGAGTCCTGAGTAACAC
AFLP_M_CCA	GATGAGTCCTGAGTAACCA
AFLP_M_CCC	GATGAGTCCTGAGTAACCC
AFLP_M_CTCA	GATGAGTCCTGAGTAACTCA
AFLP_M_CGAA	GATGAGTCCTGAGTAACGAA

### Statistical analysis

Bands were automatically recognised by GelCompar II using threshold values of 5 % of profiling (relative to the maximum value within each lane). Band matching was performed and the results were exported as a binary matrix. It was used to study the phenetic relationship among AFLP patterns by means of cluster analysis (GelCompar II) and an ordination analysis, specifically principal coordinates, using the software NTSySpc 2.11T [39]. Jaccard's similarity coefficient and the unweighted pair group method with arithmetic mean (UPGMA) were used to perform the clustering analysis. This was tested with three statistical significance tests, also using GelCompar II: the Bootstrap analysis [40] for the assessment of the robustness of dendrogram topology, the standard deviation of the cluster nodes, and cophenetic correlation as an estimation of the faithfulness of cluster analysis [41]. Firstly, bootstraping analysis was carried out, and we tried to find robust groups at the same similarity level and finally we calculated the standard deviation for these groups. Dendrogram-derived similarities were compared with experimental similarities to get cophenetic correlation.

To study the genetic structure of *Sesamum indicum *L. species, the accessions were grouped in five sets according to the geographical distribution. Gene diversity indices such as total diversity (H_T_), average diversity within group (H_ST_), diversity among groups (D_ST_) and coefficient of population differentiation (G_ST_) [42] were calculated for each band and then averaged for the total set. Heterozygocity cannot be directly observed in AFLP data because AFLP markers are dominant. To calculate allele frequencies, the absence of a band was considered as homozygous state of a recessive allele (q^2^) and presence of a band as either dominant homozygote (p^2^) or a heterozygous state (2 pq). Frequencies p and q are calculated accordingly. Also unbiased measures of genetic identity and genetic distance between groups were calculated [33]. All Nei's parameters, which use gene frequencies, were calculated using Popgene v. 1.32 software. To get another approach on the genetic structure with no assumed gene frequencies, analysis of molecular variance (AMOVA) [43] was carried out using Arlequin v. 2.000 software, to estimate variance components for the AFLP patterns and to partition the total variance into 'within groups' and 'among groups'. Significance of variance components was tested after 1000 permutations. Pairwise group F_ST _(genetic distances) values matrix was obtained to explain the significance of the variance components, also using 1000 permutations.

## Authors' contributions

HL participated in the design of the study, performed DNA extraction, AFLP and data analysis and participated in interpretation of results and manuscript preparation. PK participated in the design of the study, designed DNA extraction and participated in the discussion for preparing the manuscript and did the final revision.

## References

[B1] Bedigian D, Harlan J (1986). Evidence for cultivation on sesame in the ancient world. Economic Botany.

[B2] Ashri A (1998). Sesame breeding. Plant Breeding Reviews.

[B3] FAO (2005). FAOstat Databases.

[B4] Brar G, Ahuja R, Malik CP (1979). Sesame: its culture, genetics, breeding and biochemistry. Annu Rev Plant Sci.

[B5] Bedigian D (2003). Evolution of sesame revisited: domestication, diversity and prospects. Genetic Resources and Crop Evolution.

[B6] Zeven A, Zhukovsky P (1975). Dictionary of cultivated plants and their centres of diversity.

[B7] Hawkes J (1983). The diversity of crop plants.

[B8] Bedigian D, Smyth C, Harlan J (1986). Patterns of morphological variation in sesame. Economic Botany.

[B9] Bisht I, Mahajan R, Loknathan T, Agrawal R (1998). Diversity in Indian sesame collection and stratification of germplasm accessions in different diversity groups. Genetics Resources and Crop Evolution.

[B10] Isshiki S, Umezake T (1997). Genetic variations of isozymes in cultivated sesame. Euphytica.

[B11] Diaz A, Layrisse A, Pugh T (1999). Análisis de la diversidad genética en el ajonjolí mediante isoenzimas. Agron Trop (Maracay).

[B12] Bhat V, Babrekar P, Lakhanpaul S (1999). Study of genetic diversity in Indian and exotic sesame (*Sesamum indicum *L.) germplasm using random amplified polymorphic DNA (RAPD) markers. Euphytica.

[B13] Ercan A, Taskin M, Turgut K (2004). Analyisis of genetic diversity in Turkish sesame (*Sesamum indicum *L.) populations using RAPD markers. Genetic Resources and Crop Evolution.

[B14] Kim D, Zur G, Danin-Poleg Y, Lee S, Shim K, Kang C, Kashi Y (2002). Genetic relationships of sesame germplasm collection as revealed by inter-simple sequence repeats. Plant Breeding.

[B15] Uzun B, Lee D, Donini P, Cagrirgan M (2003). Identification of a molecular marker linked to the closed capsule mutant trait in sesame using AFLP. Plant Breeding.

[B16] Savelkoul P, Aarts H, DeHaas J, Dijkshoorn L, Duim B, Otsen M, Rademaker J, Schouls L, Lenstra J (1999). Amplified-fragment length polymorphism analysis: the state of an art. Journal of Clinical Microbiology.

[B17] Zeid M, Shon C, Link W (2003). Genetic diversity in recent elite faba bean lines using AFLP markers. Theor Appl Genet.

[B18] Fanizza G, Chaabane R, Lamaj F, Ricciardi L, Resta P (2003). AFLP analysis of genetic relationships among aromatic grapevine (*Vitis vinifera*). Theor Appl Genet.

[B19] Zong X, Kaga A, Tomooka N, Wang X, Han O, Vaughan D (2003). The genetic diversity of the *Vigna angularis *complex in Asia. Genome.

[B20] Ferriol M, Pico B, Nuez F (2003). Genetic diversity of a germplasm collection of *Cucurbita pepo *using SRAP and AFLP markers. Theor Appl Genet.

[B21] Bahulikar RA, Stanculescu D, Preston CA, Baldwin ITKatzir N (2004). ISSR and AFLP analysis of the temporal and spatial population structure of the post-fire annual, *Nicotiana attenuata*, in SW Utah. BMC Ecology.

[B22] Ude G, Pillay M, Ogundiwin E, Tenkouano A (2003). Genetic diversity in an African plantain core collection using AFLP and RAPD markers. Theor Appl Genet.

[B23] Uptmoor R, Wenzel W, Friedt W, Donaldson G, Ayisi K, Ordon F (2003). Comparative analysis on the genetic relatedness of *Sorghum bicolor *accessions from Southern Africa by RAPDs, AFLPs and SSRs. Theor Appl Genet.

[B24] Segovia-Lerma A, Cantrell R, Conway J, Ray I (2003). AFLP-based assessment of genetic diversity among nine alfalfa germplasm using bulk DNA templates. Genome.

[B25] Soleimani V, Baum B, Johnson D (2002). AFLP and pedigree-based genetic diversity estimates in modern cultivars of durum wheat [*Triticum turgidum *L. subsp. Durum (Desf.) Husn]. Theor Appl Genet.

[B26] Steiger L, Nagai C, Moore H, Morden W, Osgood V, Ming R (2002). AFLP analysis of genetic diversity within and among *Coffea arabica *cultivars. Theor Appl Genet.

[B27] Ovesná J, Poláková K, Leisová L (2002). DNA analyses and their applications in plant breeding. Czech J Genet Plant Breed.

[B28] Karp A, Kresovich S, Bhat K, Ayad W, Hodgkin T (1997). Molecular tools in plant genetic resources conservation: a guide to the technologie.

[B29] Lynch M, Milligan B (1994). Analysis of population genetic structure with RAPD markers. Molecular Ecology.

[B30] Zhyvotovsky L (1999). Estimating population structure in diploids with multilocus dominant DNA markers. Molecular Ecology.

[B31] Krauss S (2000). Accurate gene diversity estimates from amplified fragment length polymorphism (AFLP) markers. Molecular Ecology.

[B32] Caicedo A, Gaitán E, Duque M, ToroChica O, Debouck D, Tohme J (1999). AFLP fingerprinting of Phaseolus lunatus L. and related wild species from South America. Crop Science.

[B33] Nei M (1978). Estimation of average heterozygosity and genetic distance from a small number of individuals. Genetics.

[B34] Weiss E (2000). Sesame Oilseed crops.

[B35] Bedigian D, Harlan J (1983). Nuba agriculture and ethnobotany, with particular reference to sesame and sorghum. Economic Botany.

[B36] Ramanatha R, Hodgkin T (2002). Genetic diversity and conservation and utilization of plant genetic resources. Plan Cell, Tissue and Organ Culture.

[B37] Voss P, Hogers R, Bleeter M, Reijans M, van de Lee T, Hornes M, Frijters A, Pot J, Peleman J, Kuiper M, Zabeau M (1995). AFLP: a new technique for DNA fingerprinting. Nucleic Acids Res.

[B38] Reineke A, Karlovsky P (2000). Simplified AFLP protocol: replacement of primer labeling by the incoporation of alpha-labeled nucleotides during PCR. BioTechniques.

[B39] Rohlf F (2004). NTSYS-pc:numerical taxonomy and multivariate analysis system, version 211T.

[B40] Efron B (1979). Bootstrap methods: another look at the jackknife. Ann Statist.

[B41] Rohlf F, Sokal R (1981). Comparing numerical taxonomic studies. Systematic Zool.

[B42] Nei M (1987). Molecular evolutionary genetics.

[B43] Excoffier L, Smouse P, Quattro J (1992). Analysis of molecular variance inferred from metric distance among DNA haplotypes; application to human mitochondrial DNA restriction data. Genetics.

